# Crystal structure and Hirshfeld surface analysis of 1-[(benzyl­dimethyl­sil­yl)meth­yl]-1-ethyl­piperidin-1-ium ethane­sulfonate

**DOI:** 10.1107/S205698902101361X

**Published:** 2022-01-07

**Authors:** Jan-Lukas Kirchhoff, Stephan G. Koller, Kathrin Louven, Carsten Strohmann

**Affiliations:** a Technische Universität Dortmund, Fakultät Chemie und Chemische Biologie, Otto-Hahn-Strasse 6, 44227 Dortmund, Germany

**Keywords:** crystal structure, α-amino­silanes, long Si—C bonds, hydrogen bonds, Hirshfeld surface analysis

## Abstract

The title mol­ecular salt, C_17_H_30_NSi^+^·C_2_H_5_O_4_S^−^, belongs to the class of *a*-amino­silanes and was synthesized by the alkyl­ation of 1-[(benzyl­dimethyl­sil­yl)meth­yl]piperidine using diethyl sulfate. This achiral salt crystallizes in the chiral space group *P*2_1_. One of the Si—C bonds in the cation is unusually long [1.9075 (12) Å], which correlates with the adjacent quaternary N^+^ atom and was verified by quantum chemical calculations. In the crystal, the components are linked by weak C—H⋯O hydrogen bonds: a Hirshfeld surface analysis was performed to further investigate these inter­molecular inter­actions and their effects on the crystal packing.

## Abstract 2

α-Amino­silanes are distinguished by a long Si—C bond, which was confirmed in the title compound. Additionally, the supra­molecular inter­actions were determined by Hirshfeld surface analysis to investigate the influence of these contacts on the crystal packing.

## Chemical context

Selective bond transformations on silicon compounds for the cleavage of Si—C bonds are of high inter­est in silicon chemistry (Denmark *et al.*, 2007 ▸; Denmark & Liu, 2010 ▸). Compared to C—C bonds, analogous Si—C bonds can be cleaved heterolytically using strong nucleophiles (Tomooka *et al.*, 2000 ▸; Li & Hu, 2007 ▸). However, the selectivity of such reactions is limited to specific silanes. In particular, α-amino-functionalized silanes are well suited for these processes, as shown by our previous studies (Koller *et al.*, 2017 ▸). Our group has focused on using lithium organyls as strong nucleophiles to perform these Si—C transformations on highly substituted silanes (Bauer & Strohmann, 2014 ▸). In particular, derivatives of α-piperdino­benzyl­silanes have been intensively studied by our group (Strohmann *et al.*, 2004 ▸; Otte *et al.*, 2017 ▸). When strong nucleophiles are used, deprotonation in the benzyl position competes with the selective Si—C bond cleavage of the benzyl group. For this purpose, the α-amino­functionality seems to play a key role, which could be responsible for the activation of the subsequent Si—C bond cleavage. In addition, the positively charged ammonium group leads to an increased electronegativity, which enhances the electron-withdrawing effect of the substituted *α-*amino­functionality. Consequently, the *π*-character of the Si—C bond is more pronounced, leading to an elongation of the bond. Thus, a selective cleavage of the amino functionality due to the elongated Si—C bond is also conceivable (Bent, 1960 ▸, 1961 ▸; Otte *et al.*, 2017 ▸).

Several derivates of these α-piperdino­benzyl­silanes have been synthesized by our research group: 1-[(benzyl­dimethyl­sil­yl)meth­yl]-1-ethyl­piperidin-1-ium ethane­sulfonate (**1**), the title compound, represents a compound that could lead to an extension of the aforementioned Si—C bond to the nitro­gen atom *via* the quaternary ammonium cation. Structural studies concerning this type of compound should better elucidate the reactivity as well as selectivity of Si—C cleavages of the benzyl-substituted *α*-amino­silanes. 
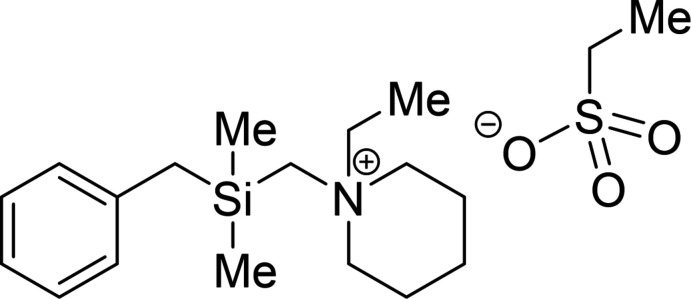


## Structural commentary

**Figure 1:**
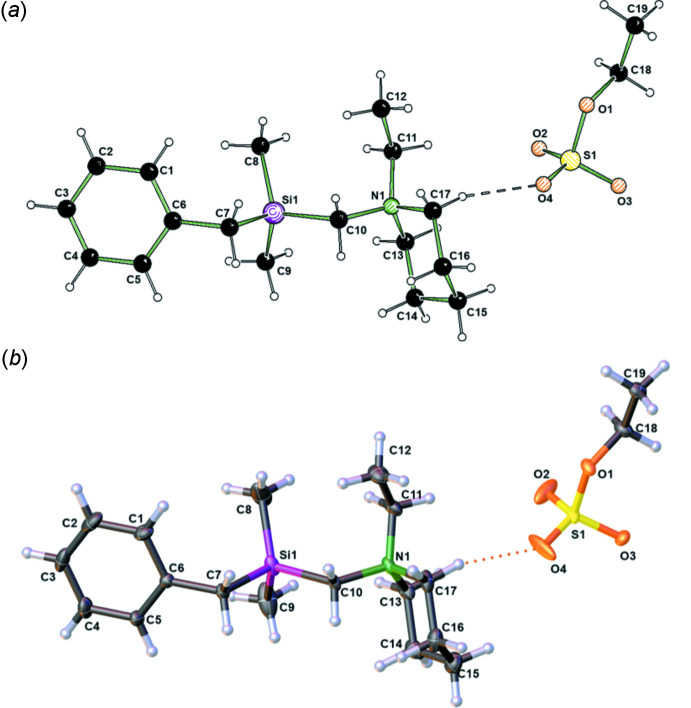
(*a*) The mol­ecular structure of **1** illustrated using *SCHAKAL99* (Keller, 1999 ▸). (*b*) The mol­ecular structure of **1** showing 50% displacement ellipsoids.

**Figure 2:**
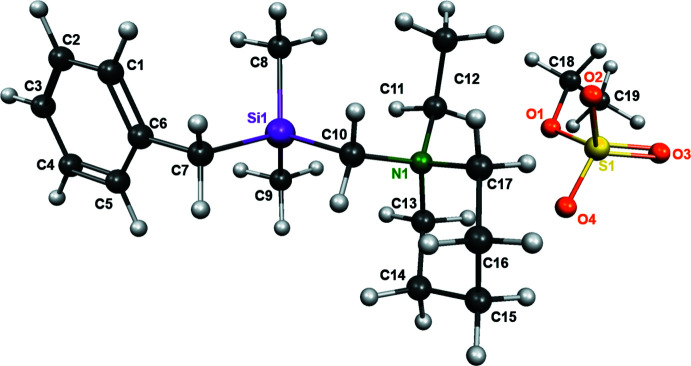
Visualization of the calculated structure of compound **1** with *Molekel 4.3* (Flükiger *et al.*, 2000 ▸) performed at the M062X/6–31+G(*d*) (Ditchfield *et al.*, 1970 ▸; Zhao & Truhlar, 2008 ▸) level.

Compound **1** crystallized from an *n*-pentane solution at 243 K in the form of colorless blocks with monoclinic (*P*2_1_) symmetry. The chiral space group indicates that the achiral compound in the elementary cell is packed chirally; the Flack absolute structure parameter amounts to −0.005 (6) (Flack, 1983 ▸). The mol­ecular structure of **1** is illustrated in Fig. 1 ▸. The Si—C bonds span the range 1.862 (2) to 1.908 (1) Å, as shown in Table 1 ▸. These values for the bond lengths are consistent with those in the literature, except for the long Si1—C10 bond length, which is related to the *α*-amino­silane functionality (Allen *et al.*, 1987 ▸). This observed elongation of the bond can be explained by the very electropositive feature of carbon atom C10. In addition, the ethyl­ated ammonium cation pushes even more electron density from C10 toward the amino functionality. There are only a few known species with such a long Si—C bond, which in turn may play a crucial role in the reactivity of α-amino-substituted silanes. Quantum chemical calculations at the M062X/6-31+G(d) level confirm the experimentally observed long Si—C bond. The calculated structure of compound **1** is shown in Fig. 2 ▸.

The silicon center in **1** features a tetra­hedral geometry, which is significantly distorted, as shown by the smallest angle of 98.35 (5)° (C7—Si1—C10) and the largest angle of 114.32 (7)° (C8—Si1—C10). This geometric distortion has been observed in many complex substituted silicon compounds and depends on the substituents (Otte *et al.*, 2017 ▸). However, the distortion is large for compound **1** compared to most known silanes (Krupp *et al.*, 2020 ▸).

## Supra­molecular features

**Figure 3:**
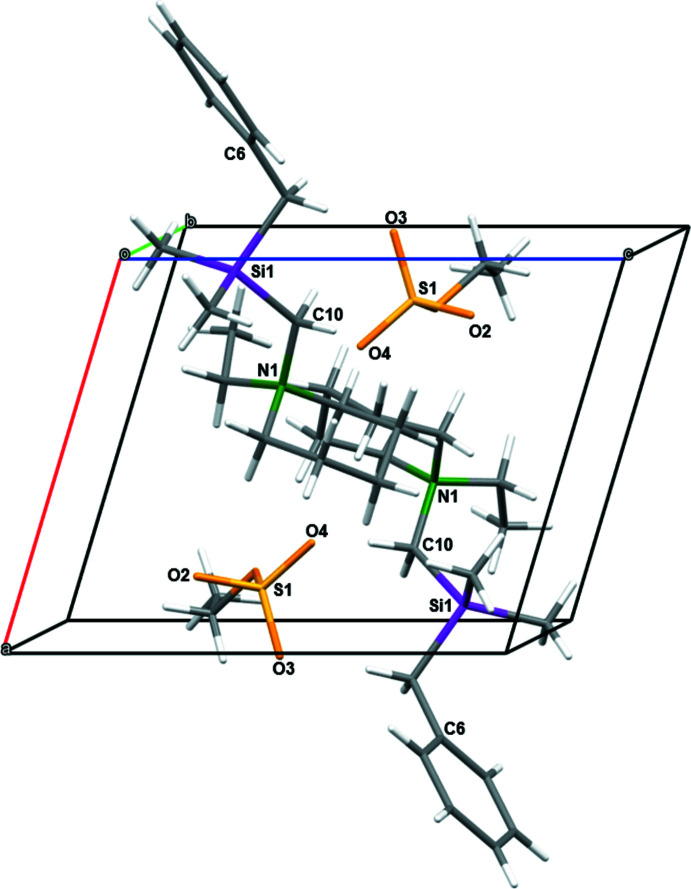
A view along the *b*-axis direction of the crystal packing of compound **1**.

**Figure 4:**
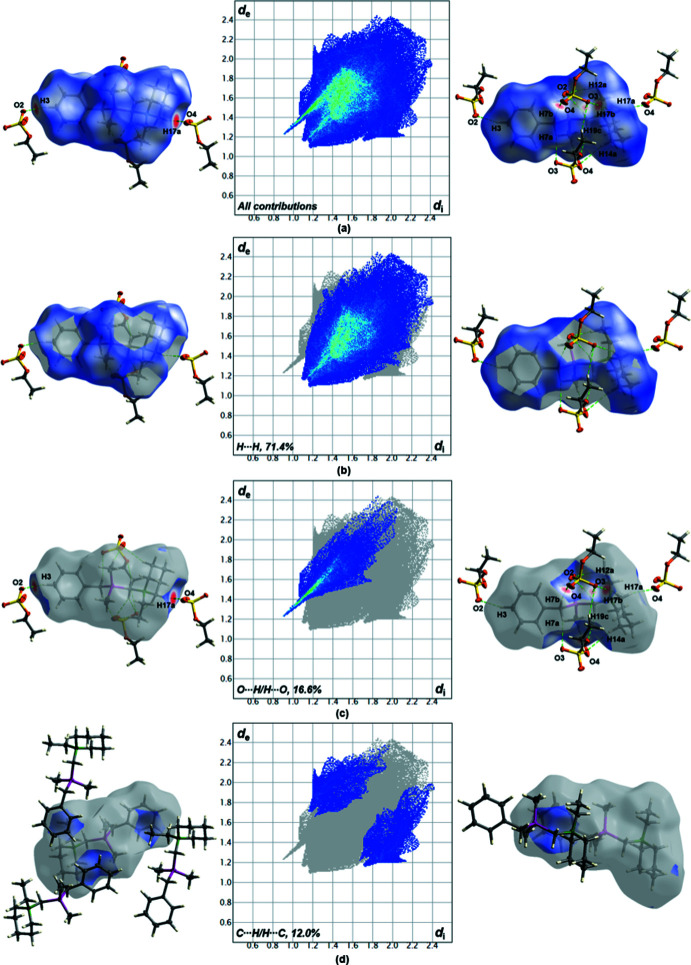
Hirshfeld surfaces and two-dimensional fingerprint plots of **1** showing close contacts for (*a*) all contributions in the crystal and (*b*) H⋯H, (*c*) O⋯H/H⋯O and (*d*) C⋯H/H⋯C inter­actions. Symmetry code: −*x*, 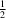 + *y*, −*z*.

The crystal packing along the *b*-axis of compound **1** is illustrated in Fig. 3 ▸. Further studies of the packing in the solid state were aimed at finding hydrogen bonds of compound **1** as well as discussing the intensities of those hydrogen bonds. These studies were performed using Hirshfeld surface analysis. The Hirshfeld surface mapped over *d*_norm_ in the range from −0.072 to 1.201 arbitrary units as well as the related fingerprints plots generated by *CrystalExplorer2021* (Spackman *et al.*, 2021 ▸, Turner *et al.*, 2017 ▸) are illustrated in Fig. 4 ▸. With a share of 71.4%, most of the inter­actions relate to weak van der Waals H⋯H contacts, which should play a minor role for the packing of the crystal. In contrast, the role of O⋯H/H⋯O contacts should be predominant in the crystal arrangement in the unit cell, as shown by the significant red spots on the Hirshfeld surface. Numerous hydrogen bonds of the ethyl sulfate group to the ammonium cation are visible on the surface. The contribution of these contacts amounts to 16.6%. C⋯H/H⋯C contacts as well as H⋯H contacts do not show as intense spots on the Hirshfeld surface and should not be considered as relevant as the O⋯H/H⋯O contacts for the crystal packing. All hydrogen bonds up to a distance of 3.4 Å as well as an angle of at least 155° are listed in Table 2 ▸. According to Perlstein (2001 ▸), all hydrogen bonds listed in Table 2 ▸ have a weak to moderately strong character, which can be explained in particular by the non-linear angles of 156 (7)° (C7—H7*B*⋯O2^ii^) to 167 (2)° (C3—H3⋯O2^i^). The shortest hydrogen-bond length is 3.1815 (16) Å and is the strongest supra­molecular inter­action with an angle of 162.8 (17)° (C17—H17*A*⋯O4). Analysis of the hydrogen-bonding network shows that all the hydrogen bonds shown in Table 2 ▸ can be assigned to one graph-set motif [*D*_1_^1^(2); Etter *et al.*, 1990 ▸] and all of these bonds are linearly connected to two different atoms.

## Database survey

There are some other examples of crystallographically characterized *α*-amino­silane derivatives that are structurally based on compound **1** and its starting compound **2**. Examples of such *α*-piperidino­silanes found in the Cambridge Structural Database (WebCSD, November 2021; Groom *et al.*, 2016 ▸) are (*R*)-1-methyl-1-{[meth­yl(phen­yl)(tri­methyl­germ­yl)sil­yl]meth­yl}piperidinium iodide, C_17_H_32_GeNSiI (CSD refcode BOFLOY; Strohmann *et al.*, 2008 ▸), (tri­phenyl­silyl­piperid­in­yl­car­bene)tetra­carbonyl­tungsten(0), C_28_H_25_NO_4_SiW (DIZWUE; Schubert *et al.*, 1986 ▸), [bis­(tri­methyl­sil­yl)meth­yl]bis­[diphen­yl(*N*-piperidino­methyl­sil­yl)meth­yl]gall­ium *n*-pentane solvate, C_45_H_67_GaN_2_Si_4_·0.5(C_5_H_12_) (MASLUN; Uhl *et al.*, 2000 ▸), 8-chloro-8,8-dimethyl-1-aza-7-oxa-8-silabi­cyclo­(4.3.0)non-6-ene, C_8_H_16_ClNOSi (FUSYIB; Macharashvili *et al.*, 1987 ▸), 1-{[benz­yl(meth­yl)phenyl­sil­yl]meth­yl}piperidinium bromide, C_20_H_28_NSiBr (NUPMUI; Barth *et al.*, 2015 ▸), *N*-(tri­phenyl­silylmeth­yl)-5,6-aza-C60fulleroid, C_79_H_17_NSi (YOXBOD; Hachiya *et al.*, 2009 ▸).

## Synthesis and crystallization

**Figure 5:**
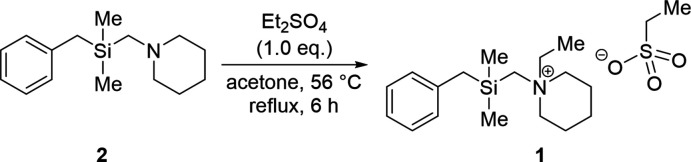
Reaction scheme of the alkyl­ation of **2** with diethyl sulfate for the synthesis of **1**.

The reaction scheme for the synthesis of **1** is illustrated in Fig. 5 ▸: 1-[(benzyl­dimethyl­sil­yl)meth­yl]piperidine (**2**) (0.81 mmol) was dissolved in acetone (3 ml) and diethyl sulfate (0.81 mmol) was added dropwise to the solution. The reaction mixture was stirred and heated for 6 h at 329 K. Afterwards the reaction was quenched by the addition of a mixture of H_2_O (2 ml) and NH_3_ (2 ml). The aqueous phase was extracted three times with CH_2_Cl_2_ and the combined organic phases were dried over Na_2_SO_4_. After the removal of volatile compounds, the raw product was dissolved in *n*-pentane (1 ml) and stored at 243 K. The title salt (**1**) was isolated as colorless crystalline blocks.

^1^H NMR (300.25 MHz, CDCl_3_): *δ* = 0.30 [*s*, 6H, Si(C*H*_3_)_2_], 1.24–1.31 (*m*, 6H, OCH_2_C*H*_3_, NCH_2_C*H*_3_), 1.65–1.90 [*br. m*, 6H, N(CH_2_CH_2_)_2_, NCH_2_CH_2_CH_2_], 2.29 (*s*, 2H, SiC*H*_2_C_ar_), 3.12 (*s*, 2H, SiC*H*_2_N), 3.37–3.56 [*br. m*, 6H, N(C*H*_2_)_3_], 4.12 (*q*, 2H, ^3^ *J*_H–H_ = 7.1Hz, OC*H*_2_CH_3_), 7.04 (*d*, 2H, ^3^ *J*_H–H_ = 7.0Hz, C*H*_ar_), 7.10–7.15 (*m*, 1H, C*H*_ar_), 7.24 (*d*, 2H, ^3^ *J*_H–H_ = 7.6Hz, C*H*_ar_) ppm.

## Refinement

Crystal data, data collection and structure refinement details are summarized in Table 3 ▸. All H atoms were refined freely using independent values for each *U*_iso_(H).

## Supplementary Material

Crystal structure: contains datablock(s) I. DOI: 10.1107/S205698902101361X/hb8003sup1.cif

Structure factors: contains datablock(s) I. DOI: 10.1107/S205698902101361X/hb8003Isup2.hkl

Supporting information file. DOI: 10.1107/S205698902101361X/hb8003Isup3.cml

CCDC reference: 2131144

Additional supporting information:  crystallographic information; 3D view; checkCIF report

## Figures and Tables

**Table 1:** Selected bond lengths (Å)

Si1—C7	1.8814 (11)	Si1—C9	1.8662 (18)
Si1—C8	1.862 (2)	Si1—C10	1.9075 (12)

**Table 2:** Hydrogen-bond geometry (Å, °)

*D*—H⋯*A*	*D*—H	H⋯*A*	*D*⋯*A*	*D*—H⋯*A*
C3—H3⋯O2^i^	0.93 (3)	2.39 (3)	3.2990 (17)	167 (2)
C7—H7*B*⋯O2^ii^	0.91 (3)	2.54 (3)	3.3881 (16)	156 (2)
C17—H17*A*⋯O4	0.95 (2)	2.26 (2)	3.1815 (16)	162.8 (17)
C17—H17*B*⋯O3^ii^	0.93 (2)	2.47 (2)	3.3680 (15)	161.3 (19)

Symmetry codes: (i) -x, y-{\script{1\over 2}}, -z; (ii) x-1, y, z.

**Table 3:** Experimental details

Crystal data	
Chemical formula	C_17_H_30_NSi^+^·C_2_H_5_O_4_S^−^
*M*_r_	401.63
Crystal system, space group	Monoclinic, *P*2_1_
Temperature (K)	100
*a*, *b*, *c* (Å)	8.4627 (8), 12.8187 (11), 10.3926 (9)
β (°)	107.033 (3)
*V* (Å^3^)	1077.95 (17)
*Z*	2
Radiation type	Mo *K*α
μ (mm^−1^)	0.23
Crystal size (mm)	0.82 × 0.44 × 0.38

Data collection	
Diffractometer	Bruker D8 VENTURE
Absorption correction	Multi-scan (*SADABS*; Bruker, 2021 ▸)
*T*_min_, *T*_max_	0.699, 0.747
No. of measured, independent and observed [*I* > 2σ(*I*)] reflections	68418, 8122, 7987
*R*_int_	0.021
(sin θ/λ)_max_ (Å^−1^)	0.766

Refinement	
*R*[*F* ^2^ > 2σ(*F* ^2^)], *wR*(*F* ^2^), *S*	0.024, 0.065, 1.05
No. of reflections	8122
No. of parameters	375
No. of restraints	1
H-atom treatment	All H-atom parameters refined
Δρ_max_, Δρ_min_ (e Å^−3^)	0.53, −0.59
Absolute structure	Flack *x* determined using 3811 quotients [(*I* ^+^)−(*I* ^−^)]/[(*I* ^+^)+(*I* ^−^)] (Parsons *et al.*, 2013 ▸)
Absolute structure parameter	−0.005 (6)

Computer programs: *APEX4* and *SAINT* (Bruker, 2021 ▸), *SHELXS* (Sheldrick, 2008 ▸), *SHELXL* (Sheldrick, 2015 ▸), *OLEX2* (Dolomanov *et al.*, 2009 ▸), *CrystalExplorer21* (Spackman *et al.*, 2021 ▸; Turner *et al.*, 2017 ▸), *publCIF* (Westrip, 2010 ▸), *Mercury* (Macrae *et al.*, 2020 ▸), *GaussView 6.016* (Frisch *et al.*, 2016 ▸), *Gaussian 09 Revision A.02* (Frisch *et al.*, 2016 ▸), *SCHAKAL99* (Keller, 1999 ▸) and *Molekel 4.3* (Flükiger *et al.*, 2000 ▸).

## supplementary crystallographic information

### Crystal data

**Table:**

C_17_H_30_NSi^+^·C_2_H_5_O_4_S^−^	*F*(000) = 436
*M**_r_* = 401.63	*D*_x_ = 1.237 Mg m^−^^3^
Monoclinic, *P*2_1_	Mo *K*α radiation, λ = 0.71073 Å
*a* = 8.4627 (8) Å	Cell parameters from 9642 reflections
*b* = 12.8187 (11) Å	θ = 3.0–30.6°
*c* = 10.3926 (9) Å	µ = 0.23 mm^−^^1^
β = 107.033 (3)°	*T* = 100 K
*V* = 1077.95 (17) Å^3^	Block, colourless
*Z* = 2	0.82 × 0.44 × 0.38 mm

### Data collection

**Table:**

Bruker D8 VENTURE diffractometer	8122 independent reflections
Radiation source: microfocus sealed X-ray tube, Incoatec Iµs	7987 reflections with *I* > 2σ(*I*)
HELIOS mirror optics monochromator	*R*_int_ = 0.021
Detector resolution: 10.4167 pixels mm^-1^	θ_max_ = 33.0°, θ_min_ = 2.5°
ω and φ scans	*h* = −12→12
Absorption correction: multi-scan (SADABS; Bruker, 2021)	*k* = −19→19
*T*_min_ = 0.699, *T*_max_ = 0.747	*l* = −15→15
68418 measured reflections	

### Refinement

**Table:**

Refinement on *F*^2^	Hydrogen site location: difference Fourier map
Least-squares matrix: full	All H-atom parameters refined
*R*[*F*^2^ > 2σ(*F*^2^)] = 0.024	*w* = 1/[σ^2^(*F*_o_^2^) + (0.039*P*)^2^ + 0.1456*P*] where *P* = (*F*_o_^2^ + 2*F*_c_^2^)/3
*wR*(*F*^2^) = 0.065	(Δ/σ)_max_ = 0.001
*S* = 1.05	Δρ_max_ = 0.53 e Å^−^^3^
8122 reflections	Δρ_min_ = −0.59 e Å^−^^3^
375 parameters	Absolute structure: Flack *x* determined using 3811 quotients [(*I*^+^)-(*I*^-^)]/[(*I*^+^)+(*I*^-^)] (Parsons *et al.*, 2013)
1 restraint	Absolute structure parameter: −0.005 (6)
Primary atom site location: iterative	

### Special details

**Table:**

Geometry. All esds (except the esd in the dihedral angle between two l.s. planes) are estimated using the full covariance matrix. The cell esds are taken into account individually in the estimation of esds in distances, angles and torsion angles; correlations between esds in cell parameters are only used when they are defined by crystal symmetry. An approximate (isotropic) treatment of cell esds is used for estimating esds involving l.s. planes.

### Fractional atomic coordinates and isotropic or equivalent isotropic displacement parameters (Å^2^)

**Table:**

	*x*	*y*	*z*	*U*_iso_*/*U*_eq_	
S1	0.88614 (3)	0.62026 (2)	0.40720 (3)	0.01913 (6)	
Si1	0.05612 (4)	0.31084 (3)	0.20000 (3)	0.01987 (7)	
O3	1.05747 (10)	0.61067 (7)	0.48491 (8)	0.01886 (15)	
O1	0.85485 (10)	0.74269 (6)	0.37242 (10)	0.01822 (15)	
O4	0.77064 (16)	0.60053 (10)	0.4817 (2)	0.0533 (5)	
O2	0.85245 (18)	0.56667 (8)	0.27863 (13)	0.0439 (4)	
N1	0.35820 (12)	0.43767 (7)	0.34820 (11)	0.01724 (16)	
C10	0.19151 (13)	0.39252 (8)	0.34376 (12)	0.01601 (18)	
C14	0.53245 (16)	0.29189 (10)	0.48490 (16)	0.0253 (2)	
C17	0.41837 (15)	0.50777 (10)	0.47118 (15)	0.0230 (2)	
C6	−0.27742 (13)	0.25033 (8)	0.16162 (10)	0.01469 (17)	
C5	−0.31035 (14)	0.14496 (9)	0.17585 (11)	0.01782 (18)	
C13	0.48527 (15)	0.35320 (9)	0.35398 (14)	0.0211 (2)	
C7	−0.13320 (13)	0.30314 (9)	0.25953 (11)	0.01589 (17)	
C1	−0.38217 (16)	0.30517 (11)	0.05336 (13)	0.0247 (2)	
C15	0.59552 (18)	0.36517 (13)	0.60478 (17)	0.0298 (3)	
C18	0.96437 (15)	0.78813 (9)	0.30464 (13)	0.0206 (2)	
C4	−0.44312 (17)	0.09575 (11)	0.08387 (14)	0.0258 (2)	
C3	−0.54609 (16)	0.15050 (14)	−0.02339 (13)	0.0295 (3)	
C16	0.46575 (16)	0.44744 (12)	0.60249 (15)	0.0276 (3)	
C11	0.35005 (16)	0.50178 (10)	0.22355 (15)	0.0245 (2)	
C19	0.93127 (16)	0.90390 (9)	0.29232 (12)	0.0202 (2)	
C12	0.23198 (19)	0.59372 (11)	0.19959 (18)	0.0306 (3)	
C8	0.0020 (3)	0.3761 (2)	0.03268 (16)	0.0472 (5)	
C2	−0.51474 (18)	0.25531 (15)	−0.03823 (14)	0.0316 (3)	
C9	0.1422 (2)	0.17830 (15)	0.1885 (3)	0.0455 (5)	
H13A	0.442 (2)	0.3078 (18)	0.274 (2)	0.024 (4)*	
H3	−0.638 (3)	0.121 (2)	−0.085 (3)	0.043 (6)*	
H13B	0.583 (3)	0.3899 (17)	0.346 (2)	0.025 (5)*	
H5	−0.241 (2)	0.1076 (17)	0.250 (2)	0.024 (4)*	
H11A	0.318 (2)	0.4547 (16)	0.1467 (19)	0.017 (4)*	
H12A	0.122 (3)	0.5740 (19)	0.193 (2)	0.029 (5)*	
H10A	0.128 (3)	0.4494 (18)	0.358 (2)	0.026 (5)*	
H7A	−0.108 (3)	0.2671 (17)	0.343 (2)	0.023 (4)*	
H16A	0.368 (3)	0.4130 (18)	0.614 (2)	0.032 (5)*	
H7B	−0.162 (3)	0.369 (2)	0.277 (3)	0.041 (6)*	
H8A	−0.071 (4)	0.332 (3)	−0.020 (3)	0.056 (8)*	
H18A	1.077 (3)	0.7716 (19)	0.355 (2)	0.037 (6)*	
H17A	0.512 (3)	0.5403 (16)	0.456 (2)	0.023 (5)*	
H16B	0.512 (3)	0.504 (2)	0.676 (3)	0.040 (6)*	
H17B	0.331 (3)	0.5512 (18)	0.475 (2)	0.032 (5)*	
H12B	0.233 (3)	0.630 (2)	0.106 (3)	0.043 (6)*	
H2	−0.586 (3)	0.296 (2)	−0.110 (2)	0.041 (6)*	
H11B	0.454 (3)	0.525 (2)	0.235 (2)	0.033 (6)*	
H14A	0.439 (3)	0.2515 (19)	0.494 (2)	0.030 (5)*	
H14B	0.617 (3)	0.2459 (19)	0.477 (2)	0.033 (6)*	
H19A	0.825 (3)	0.916 (2)	0.235 (3)	0.040 (6)*	
H19B	0.945 (3)	0.933 (2)	0.382 (2)	0.037 (6)*	
H9A	0.058 (3)	0.133 (2)	0.123 (3)	0.048 (7)*	
H19C	1.009 (3)	0.943 (2)	0.251 (2)	0.031 (5)*	
H1	−0.362 (3)	0.379 (2)	0.045 (3)	0.041 (6)*	
H10B	0.205 (3)	0.3469 (18)	0.418 (2)	0.027 (5)*	
H9B	0.231 (5)	0.175 (4)	0.154 (4)	0.090 (13)*	
H4	−0.477 (3)	0.018 (2)	0.093 (3)	0.037 (6)*	
H8B	0.103 (6)	0.397 (4)	0.005 (4)	0.095 (14)*	
H18B	0.945 (3)	0.751 (2)	0.216 (2)	0.037 (6)*	
H15A	0.700 (4)	0.397 (2)	0.597 (3)	0.058 (8)*	
H12C	0.274 (3)	0.647 (2)	0.270 (3)	0.046 (7)*	
H15B	0.623 (4)	0.327 (2)	0.686 (3)	0.051 (7)*	
H8C	−0.044 (5)	0.439 (4)	0.038 (4)	0.078 (11)*	
H9C	0.167 (5)	0.140 (4)	0.293 (4)	0.092 (12)*	

### Atomic displacement parameters (Å^2^)

**Table:**

	*U*^11^	*U*^22^	*U*^33^	*U*^12^	*U*^13^	*U*^23^
S1	0.01109 (10)	0.01196 (10)	0.03141 (13)	0.00019 (8)	0.00167 (8)	0.00310 (9)
Si1	0.01993 (14)	0.02043 (14)	0.02339 (14)	−0.00530 (11)	0.01279 (11)	−0.00846 (11)
O3	0.0134 (3)	0.0214 (4)	0.0193 (3)	0.0031 (3)	0.0009 (3)	0.0034 (3)
O1	0.0140 (3)	0.0118 (3)	0.0301 (4)	0.0013 (3)	0.0084 (3)	0.0021 (3)
O4	0.0297 (5)	0.0335 (7)	0.1128 (13)	0.0101 (5)	0.0458 (7)	0.0345 (7)
O2	0.0507 (7)	0.0172 (4)	0.0404 (6)	0.0058 (4)	−0.0234 (5)	−0.0085 (4)
N1	0.0133 (4)	0.0120 (4)	0.0294 (5)	−0.0001 (3)	0.0109 (3)	−0.0006 (3)
C10	0.0127 (4)	0.0146 (4)	0.0237 (5)	−0.0019 (3)	0.0099 (3)	−0.0023 (3)
C14	0.0194 (5)	0.0169 (5)	0.0405 (7)	0.0031 (4)	0.0104 (5)	0.0045 (4)
C17	0.0145 (4)	0.0161 (5)	0.0387 (6)	−0.0020 (4)	0.0082 (4)	−0.0083 (4)
C6	0.0134 (4)	0.0151 (4)	0.0158 (4)	0.0024 (3)	0.0045 (3)	−0.0004 (3)
C5	0.0185 (4)	0.0157 (4)	0.0178 (4)	−0.0026 (3)	0.0030 (3)	−0.0012 (3)
C13	0.0168 (4)	0.0148 (4)	0.0361 (6)	0.0035 (4)	0.0148 (4)	−0.0004 (4)
C7	0.0156 (4)	0.0146 (4)	0.0185 (4)	−0.0018 (3)	0.0066 (3)	−0.0030 (3)
C1	0.0215 (5)	0.0259 (5)	0.0243 (5)	0.0078 (4)	0.0032 (4)	0.0072 (5)
C15	0.0190 (5)	0.0313 (7)	0.0367 (7)	0.0027 (5)	0.0047 (5)	0.0017 (5)
C18	0.0222 (5)	0.0148 (4)	0.0282 (5)	0.0033 (4)	0.0129 (4)	0.0036 (4)
C4	0.0235 (5)	0.0286 (6)	0.0240 (5)	−0.0094 (4)	0.0052 (4)	−0.0081 (4)
C3	0.0162 (5)	0.0506 (8)	0.0197 (5)	−0.0043 (5)	0.0022 (4)	−0.0102 (5)
C16	0.0177 (5)	0.0326 (6)	0.0321 (6)	−0.0019 (5)	0.0064 (4)	−0.0093 (5)
C11	0.0217 (5)	0.0200 (5)	0.0367 (6)	0.0007 (4)	0.0163 (5)	0.0072 (5)
C19	0.0250 (5)	0.0131 (4)	0.0230 (5)	−0.0009 (4)	0.0081 (4)	−0.0012 (4)
C12	0.0283 (6)	0.0195 (5)	0.0444 (8)	0.0042 (4)	0.0115 (6)	0.0082 (5)
C8	0.0582 (11)	0.0667 (13)	0.0200 (6)	−0.0352 (11)	0.0163 (7)	−0.0063 (7)
C2	0.0201 (5)	0.0496 (9)	0.0211 (5)	0.0105 (6)	−0.0004 (4)	0.0041 (5)
C9	0.0296 (7)	0.0320 (7)	0.0824 (14)	−0.0062 (6)	0.0279 (9)	−0.0332 (9)

### Geometric parameters (Å, º)

**Table:**

S1—O3	1.4435 (8)	C1—C2	1.396 (2)
S1—O1	1.6149 (9)	C1—H1	0.98 (3)
S1—O4	1.4364 (12)	C15—C16	1.518 (2)
S1—O2	1.4545 (13)	C15—H15A	1.00 (3)
Si1—C7	1.8814 (11)	C15—H15B	0.95 (3)
Si1—C8	1.862 (2)	C18—C19	1.5086 (16)
Si1—C9	1.8662 (18)	C18—H18A	0.97 (3)
Si1—C10	1.9075 (12)	C18—H18B	1.00 (3)
O1—C18	1.4412 (14)	C4—C3	1.388 (2)
N1—C10	1.5130 (14)	C4—H4	1.05 (3)
N1—C17	1.5227 (17)	C3—C2	1.387 (3)
N1—C13	1.5148 (15)	C3—H3	0.93 (3)
N1—C11	1.5186 (17)	C16—H16A	0.98 (2)
C10—H10A	0.94 (2)	C16—H16B	1.04 (3)
C10—H10B	0.95 (2)	C11—C12	1.5177 (19)
C14—C13	1.5199 (19)	C11—H11A	0.97 (2)
C14—C15	1.526 (2)	C11—H11B	0.91 (2)
C14—H14A	0.97 (2)	C19—H19A	0.94 (3)
C14—H14B	0.95 (2)	C19—H19B	0.99 (2)
C17—C16	1.517 (2)	C19—H19C	1.01 (2)
C17—H17A	0.95 (2)	C12—H12A	0.94 (2)
C17—H17B	0.93 (2)	C12—H12B	1.08 (3)
C6—C5	1.3957 (15)	C12—H12C	0.99 (3)
C6—C7	1.5019 (15)	C8—H8A	0.89 (3)
C6—C1	1.4005 (16)	C8—H8B	1.01 (5)
C5—C4	1.3946 (16)	C8—H8C	0.91 (4)
C5—H5	0.95 (2)	C2—H2	0.96 (3)
C13—H13A	0.99 (2)	C9—H9A	1.01 (3)
C13—H13B	0.97 (2)	C9—H9B	0.92 (4)
C7—H7A	0.95 (2)	C9—H9C	1.15 (4)
C7—H7B	0.91 (3)		
			
O3—S1—O1	106.26 (5)	C14—C15—H15A	106.7 (18)
O3—S1—O2	111.61 (7)	C14—C15—H15B	110.4 (18)
O4—S1—O3	114.45 (8)	C16—C15—C14	109.66 (11)
O4—S1—O1	101.47 (6)	C16—C15—H15A	111.9 (18)
O4—S1—O2	115.54 (11)	C16—C15—H15B	111.2 (18)
O2—S1—O1	106.15 (6)	H15A—C15—H15B	107 (3)
C7—Si1—C10	98.35 (5)	O1—C18—C19	108.00 (9)
C8—Si1—C10	114.32 (7)	O1—C18—H18A	108.5 (14)
C8—Si1—C7	109.31 (9)	O1—C18—H18B	107.5 (15)
C8—Si1—C9	110.06 (12)	C19—C18—H18A	112.9 (15)
C9—Si1—C10	113.19 (8)	C19—C18—H18B	114.0 (15)
C9—Si1—C7	111.06 (7)	H18A—C18—H18B	106 (2)
C18—O1—S1	114.55 (7)	C5—C4—H4	123.5 (14)
C10—N1—C17	109.32 (9)	C3—C4—C5	120.81 (13)
C10—N1—C13	111.87 (9)	C3—C4—H4	115.5 (14)
C10—N1—C11	111.84 (9)	C4—C3—H3	123.5 (18)
C13—N1—C17	109.24 (10)	C2—C3—C4	118.93 (12)
C13—N1—C11	105.96 (9)	C2—C3—H3	117.5 (18)
C11—N1—C17	108.49 (10)	C17—C16—C15	111.58 (12)
Si1—C10—H10A	107.8 (13)	C17—C16—H16A	109.3 (13)
Si1—C10—H10B	101.7 (14)	C17—C16—H16B	104.1 (15)
N1—C10—Si1	125.08 (8)	C15—C16—H16A	108.7 (14)
N1—C10—H10A	105.8 (14)	C15—C16—H16B	110.8 (15)
N1—C10—H10B	108.7 (13)	H16A—C16—H16B	112.3 (19)
H10A—C10—H10B	106.6 (18)	N1—C11—H11A	107.2 (12)
C13—C14—C15	110.47 (11)	N1—C11—H11B	105.7 (15)
C13—C14—H14A	110.8 (14)	C12—C11—N1	115.05 (11)
C13—C14—H14B	104.5 (14)	C12—C11—H11A	109.7 (12)
C15—C14—H14A	110.4 (14)	C12—C11—H11B	109.4 (16)
C15—C14—H14B	111.0 (15)	H11A—C11—H11B	109.7 (19)
H14A—C14—H14B	110 (2)	C18—C19—H19A	109.5 (16)
N1—C17—H17A	101.9 (12)	C18—C19—H19B	109.3 (15)
N1—C17—H17B	108.3 (14)	C18—C19—H19C	113.5 (14)
C16—C17—N1	112.92 (10)	H19A—C19—H19B	111 (2)
C16—C17—H17A	111.3 (13)	H19A—C19—H19C	106 (2)
C16—C17—H17B	105.7 (14)	H19B—C19—H19C	107 (2)
H17A—C17—H17B	117 (2)	C11—C12—H12A	112.9 (14)
C5—C6—C7	120.82 (10)	C11—C12—H12B	107.8 (14)
C5—C6—C1	118.15 (11)	C11—C12—H12C	109.8 (16)
C1—C6—C7	121.03 (10)	H12A—C12—H12B	108.6 (19)
C6—C5—H5	118.5 (13)	H12A—C12—H12C	112 (2)
C4—C5—C6	120.72 (11)	H12B—C12—H12C	106 (2)
C4—C5—H5	120.8 (13)	Si1—C8—H8A	103 (2)
N1—C13—C14	113.73 (10)	Si1—C8—H8B	113 (3)
N1—C13—H13A	107.5 (12)	Si1—C8—H8C	110 (2)
N1—C13—H13B	105.1 (13)	H8A—C8—H8B	118 (3)
C14—C13—H13A	112.5 (13)	H8A—C8—H8C	112 (3)
C14—C13—H13B	108.6 (13)	H8B—C8—H8C	101 (3)
H13A—C13—H13B	109.1 (17)	C1—C2—H2	118.4 (17)
Si1—C7—H7A	109.8 (13)	C3—C2—C1	120.64 (13)
Si1—C7—H7B	108.6 (17)	C3—C2—H2	121.0 (17)
C6—C7—Si1	113.71 (7)	Si1—C9—H9A	110.7 (16)
C6—C7—H7A	108.8 (13)	Si1—C9—H9B	116 (3)
C6—C7—H7B	109.9 (17)	Si1—C9—H9C	107 (2)
H7A—C7—H7B	106 (2)	H9A—C9—H9B	102 (3)
C6—C1—H1	118.4 (16)	H9A—C9—H9C	107 (3)
C2—C1—C6	120.75 (13)	H9B—C9—H9C	114 (3)
C2—C1—H1	120.8 (16)		
			
S1—O1—C18—C19	−174.27 (8)	C5—C4—C3—C2	0.2 (2)
O3—S1—O1—C18	55.64 (10)	C13—N1—C10—Si1	−65.17 (13)
O4—S1—O1—C18	175.59 (11)	C13—N1—C17—C16	−52.78 (12)
O2—S1—O1—C18	−63.30 (11)	C13—N1—C11—C12	−178.50 (12)
N1—C17—C16—C15	56.11 (14)	C13—C14—C15—C16	56.25 (15)
C10—Si1—C7—C6	174.81 (8)	C7—C6—C5—C4	−179.20 (11)
C10—N1—C17—C16	69.94 (12)	C7—C6—C1—C2	179.19 (11)
C10—N1—C13—C14	−67.70 (13)	C1—C6—C5—C4	0.58 (17)
C10—N1—C11—C12	59.35 (15)	C1—C6—C7—Si1	−82.81 (12)
C14—C15—C16—C17	−56.65 (16)	C15—C14—C13—N1	−56.46 (14)
C17—N1—C10—Si1	173.69 (8)	C4—C3—C2—C1	−0.2 (2)
C17—N1—C13—C14	53.48 (13)	C11—N1—C10—Si1	53.52 (12)
C17—N1—C11—C12	−61.30 (14)	C11—N1—C17—C16	−167.85 (10)
C6—C5—C4—C3	−0.40 (19)	C11—N1—C13—C14	170.17 (10)
C6—C1—C2—C3	0.4 (2)	C8—Si1—C7—C6	55.31 (11)
C5—C6—C7—Si1	96.96 (11)	C9—Si1—C7—C6	−66.31 (12)
C5—C6—C1—C2	−0.59 (19)		

### Hydrogen-bond geometry (Å, º)

**Table:**

*D*—H···*A*	*D*—H	H···*A*	*D*···*A*	*D*—H···*A*
C3—H3···O2^i^	0.93 (3)	2.39 (3)	3.2990 (17)	167 (2)
C7—H7*B*···O2^ii^	0.91 (3)	2.54 (3)	3.3881 (16)	156 (2)
C17—H17*A*···O4	0.95 (2)	2.26 (2)	3.1815 (16)	162.8 (17)
C17—H17*B*···O3^ii^	0.93 (2)	2.47 (2)	3.3680 (15)	161.3 (19)

Symmetry codes: (i) −*x*, *y*−1/2, −*z*; (ii) *x*−1, *y*, *z*.
