# Crystal structure and Hirshfeld surface analysis of 1-[(benzyl­dimethyl­sil­yl)meth­yl]-1-ethyl­piperidin-1-ium ethane­sulfonate. Corrigendum

**DOI:** 10.1107/S2056989025006012

**Published:** 2025-07-31

**Authors:** Jan-Lukas Kirchhoff, Stephan G. Koller, Kathrin Louven, Carsten Strohmann

**Affiliations:** a Technische Universität Dortmund, Fakultät Chemie und Chemische Biologie, Otto-Hahn-Strasse 6, 44227 Dortmund, Germany

**Keywords:** crystal structure, α-amino­silanes, long Si—C bonds, hydrogen bonds, Hirshfeld surface analysis

## Abstract

In the paper by Kirchhoff *et al.* [*Acta Cryst.* (2022), E**78**, 135–139], there was an error in the chemical name of the title compound.

## Abstract 2

Corrigendum to *Acta Cryst.* (2022), E**78**, 135–139.

The chemical name of the title compound in the paper by Kirchhoff *et al.* (2022 ▸) is incorrect and should be ‘1-[(benzyl­dimethyl­sil­yl)meth­yl]-1-ethyl­piperidin-1-ium ethane­sulfate’. The original article has been updated and is available as supporting information. Specifically, the title and the occurrence of the chemical name in the *Chemical context* section have been updated. The chemical scheme and Fig. 5 have been modified to show the correct structure.

## Supplementary Material

Crystal structure: contains datablock(s) I. DOI: 10.1107/S2056989025006012/me6338sup1.cif

Updated PDF of original article. DOI: 10.1107/S2056989025006012/me6338sup2.pdf
