# Supplementary material for: Crystal structure and Hirshfeld surface analysis of 1-[(benzyl­dimethyl­sil­yl)meth­yl]-1-ethyl­piperidin-1-ium ethane­sulfonate. Corrigendum
Source: Acta Crystallogr E Crystallogr Commun. 2025 Jul 31;81(Pt 8):782. doi: 10.1107/S2056989025006012 (PMC12326504; doi:10.1107/S2056989025006012)
Supplement: Supplementary file 2 [file e-81-00782-sup2.pdf]

ARTICLE IN PRESS – Acta Cryst. E

Acta Cryst E
CRYSTALLOGRAPHIC COMMUNICATIONS

ISSN 2056-9890

Crystal structure and Hirshfeld surface analysis of 1-[(benzyldimethylsilyl)methyl]-1-ethylpiperidin-1-ium ethanesulfate

Proof instructions

Proof corrections should be returned by **7 July 2025**. After this period, the Editors reserve the right to publish your article with only the Managing Editor's corrections.

Please
(1) Read these proofs and assess whether any corrections are necessary.
(2) Check that any technical editing queries highlighted in **bold underlined** text have been answered.
(3) Send corrections by email to **checkin@iucr.org**. Please describe corrections using plain text, where possible, giving the line numbers indicated in the proof. Please do not make corrections to the pdf file electronically and please do not return the pdf file. If no corrections are required please let us know.

Please check the following details for your article:

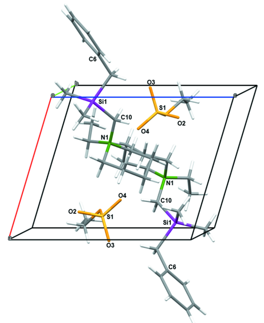

Article thumbnail

Synopsis: *α*-Aminosilanes are distinguished by a long Si—C bond, which was confirmed in the title compound. Additionally, the supramolecular interactions were determined by Hirshfeld surface analysis to investigate the influence of these contacts on the crystal packing.

Abbreviated author list: Kirchhoff, J.-L.; Koller, S.G.; Louven, K.; Strohmann, C.

Keywords: crystal structure, *α*-aminosilanes, long Si—C bonds, hydrogen bonds, Hirshfeld surface analysis

How to cite your article

Your article has not yet been assigned page numbers, but may be cited using the doi:

J.-L. Kirchhoff, S.G. Koller, K. Louven and C. Strohmann (2022). *Acta Cryst.* E**78**, https://doi.org/10.1107/S2056989025006012.

When the final version of your article is published, you will be sent the full citation details and will be given instructions on how to download an electronic reprint.

Acta Cryst E
CRYSTALLOGRAPHIC COMMUNICATIONS

ISSN 2056-9890

Received 29 November 2021
Accepted 27 December 2021

Edited by W. T. A. Harrison, University of Aberdeen, Scotland

Keywords: crystal structure; α-aminosilanes; long Si—C bonds; hydrogen bonds; Hirshfeld surface analysis.

CCDC reference: 2131144

Supporting information: this article has supporting information at journals.iucr.org/e

Crystal structure and Hirshfeld surface analysis of 1-[(benzyldimethylsilyl)methyl]-1-ethylpiperidin-1-ium ethanesulfate

Jan-Lukas Kirchhoff, Stephan G. Koller, Kathrin Louven and Carsten Strohmann*

Technische Universität Dortmund, Fakultät Chemie und Chemische Biologie, Otto-Hahn-Strasse 6, 44227 Dortmund, Germany. *Correspondence e-mail: carsten.strohmann@tu-dortmund.de

The title molecular salt, $C_{17}H_{30}NSi^{+}\cdot C_2H_5O_4S^{-}$, belongs to the class of *α*-aminosilanes and was synthesized by the alkylation of 1-[(benzyldimethylsilyl)methyl]piperidine using diethyl sulfate. This achiral salt crystallizes in the chiral space group $P2_1$. One of the Si—C bonds in the cation is unusually long [1.9075 (12) Å], which correlates with the adjacent quaternary N^{+} atom and was verified by quantum chemical calculations. In the crystal, the components are linked by weak C—H···O hydrogen bonds: a Hirshfeld surface analysis was performed to further investigate these intermolecular interactions and their effects on the crystal packing.

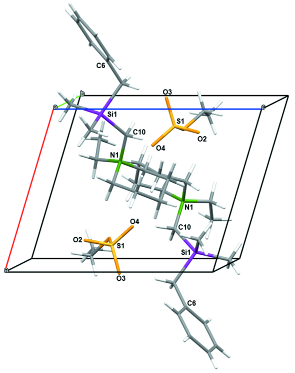

OPEN ACCESS
Published under a CC BY 4.0 licence

1. Chemical context

Selective bond transformations on silicon compounds for the cleavage of Si—C bonds are of high interest in silicon chemistry (Denmark *et al.*, 2007; Denmark & Liu, 2010). Compared to C—C bonds, analogous Si—C bonds can be cleaved heterolytically using strong nucleophiles (Tomooka *et al.*, 2000; Li & Hu, 2007). However, the selectivity of such reactions is limited to specific silanes. In particular, *α*-amino-functionalized silanes are well suited for these processes, as shown by our previous studies (Koller *et al.*, 2017). Our group has focused on using lithium organyls as strong nucleophiles to perform these Si—C transformations on highly substituted silanes (Bauer & Strohmann, 2014). In particular, derivatives of *α*-piperdinobenzylsilanes have been intensively studied by our group (Strohmann *et al.*, 2004; Otte *et al.*, 2017). When strong nucleophiles are used, deprotonation in the benzyl position competes with the selective Si—C bond cleavage of the benzyl group. For this purpose, the *α*-aminofunctionality seems to play a key role, which could be responsible for the activation of the subsequent Si—C bond cleavage. In addition, the positively charged ammonium group leads to an increased electronegativity, which enhances the electron-withdrawing effect of the substituted *α*-aminofunctionality. Consequently, the *π*-character of the Si—C bond is more pronounced, leading to an elongation of the bond. Thus, a selective cleavage of the amino functionality due to the elongated Si—C bond is also conceivable (Bent, 1960, 1961; Otte *et al.*, 2017).

Several derivates of these *α*-piperdinobenzylsilanes have been synthesized by our research group: 1-[(benzyldimethylsilyl)methyl]-1-ethylpiperidin-1-ium ethanesulfate (**1**), the title compound, represents a compound that could lead to an extension of the aforementioned Si—C bond to the nitrogen

Table 1
Selected bond lengths (Å).

Si1—C7	1.8814 (11)	Si1—C9	1.8662 (18)
Si1—C8	1.862 (2)	Si1—C10	1.9075 (12)

atom *via* the quaternary ammonium cation. Structural studies concerning this type of compound should better elucidate the reactivity as well as selectivity of Si—C cleavages of the benzyl-substituted α-aminosilanes.

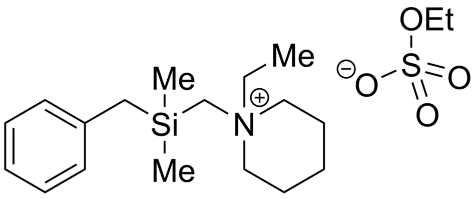

2. Structural commentary

Compound **1** crystallized from an *n*-pentane solution at 243 K in the form of colorless blocks with monoclinic ($P2_1$) symmetry. The chiral space group indicates that the achiral compound in the elementary cell is packed chirally; the Flack absolute structure parameter amounts to −0.005 (6) (Flack, 1983). The molecular structure of **1** is illustrated in Fig. 1. The Si—C bonds span the range 1.862 (2) to 1.908 (1) Å, as shown in Table 1. These values for the bond lengths are consistent with those in the literature, except for the long Si1—C10 bond length, which is related to the α-aminosilane functionality (Allen *et al.*, 1987). This observed elongation of the bond can be explained by the very electropositive feature of carbon atom C10. In addition, the ethylated ammonium cation pushes even more electron density from C10 toward the amino functionality. There are only a few known species with such a long Si—C bond, which in turn may play a crucial role in the reactivity of α-amino-substituted silanes. Quantum chemical calculations at the M062X/6-31+G(d) level confirm the experimentally observed long Si—C bond. The calculated structure of compound **1** is shown in Fig. 2.

The silicon center in **1** features a tetrahedral geometry, which is significantly distorted, as shown by the smallest angle of 98.35 (5)° (C7—Si1—C10) and the largest angle of 114.32 (7)° (C8—Si1—C10). This geometric distortion has been observed in many complex substituted silicon compounds and depends on the substituents (Otte *et al.*, 2017). However, the distortion is large for compound **1** compared to most known silanes (Krupp *et al.*, 2020).

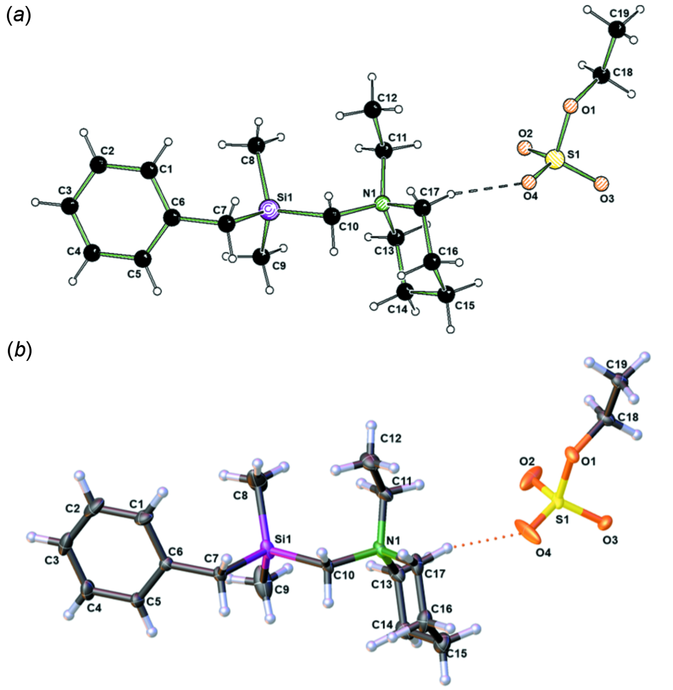

Figure 1
(*a*) The molecular structure of **1** illustrated using *SCHAKAL99* (Keller, 1999). (*b*) The molecular structure of **1** showing 50% displacement ellipsoids.

3. Supramolecular features

The crystal packing along the *b*-axis of compound **1** is illustrated in Fig. 3. Further studies of the packing in the solid state were aimed at finding hydrogen bonds of compound **1** as well as discussing the intensities of those hydrogen bonds. These studies were performed using Hirshfeld surface analysis. The Hirshfeld surface mapped over d_{norm} in the range from −0.072 to 1.201 arbitrary units as well as the related fingerprints plots generated by *CrystalExplorer2021* (Spackman *et al.*, 2021, Turner *et al.*, 2017) are illustrated in Fig. 4. With a share of 71.4%, most of the interactions relate to weak van der Waals H···H contacts, which should play a minor role for the packing of the crystal. In contrast, the role of O···H/H···O contacts should be predominant in the crystal arrangement in the unit cell, as shown by the significant red spots on the Hirshfeld surface. Numerous hydrogen bonds of the ethyl sulfate group to the ammonium cation are visible on the surface. The contribution of these contacts amounts to 16.6%. C···H/H···C contacts as well as H···H contacts do not

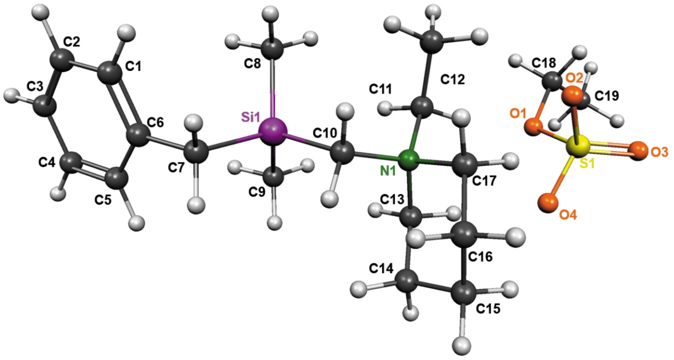

Figure 2
Visualization of the calculated structure of compound **1** with *Molekel 4.3* (Flükiger *et al.*, 2000) performed at the M062X/6–31+G(*d*) (Ditchfield *et al.*, 1970; Zhao & Truhlar, 2008) level.

Table 2
Hydrogen-bond geometry (Å, °).

D—H···A	D—H	H···A	D···A	D—H···A
C3—H3···O2[i]	0.93 (3)	2.39 (3)	3.2990 (17)	167 (2)
C7—H7B···O2[ii]	0.91 (3)	2.54 (3)	3.3881 (16)	156 (2)
C17—H17A···O4	0.95 (2)	2.26 (2)	3.1815 (16)	162.8 (17)
C17—H17B···O3[ii]	0.93 (2)	2.47 (2)	3.3680 (15)	161.3 (19)

Symmetry codes: (i) $-x, y-\frac{1}{2}, -z$; (ii) $x-1, y, z$.

show as intense spots on the Hirshfeld surface and should not be considered as relevant as the O···H/H···O contacts for the crystal packing. All hydrogen bonds up to a distance of 3.4 Å as well as an angle of at least 155° are listed in Table 2. According to Perlstein (2001), all hydrogen bonds listed in Table 2 have a weak to moderately strong character, which can be explained in particular by the non-linear angles of 156 (7)° (C7—H7*B*···O2[ii]) to 167 (2)° (C3—H3···O2[i]). The shortest hydrogen-bond length is 3.1815 (16) Å and is the strongest supramolecular interaction with an angle of 162.8 (17)° (C17—H17*A*···O4). Analysis of the hydrogen-bonding network shows that all the hydrogen bonds shown in Table 2 can be assigned to one graph-set motif [$D_1^1(2)$; Etter *et al.*, 1990] and all of these bonds are linearly connected to two different atoms.

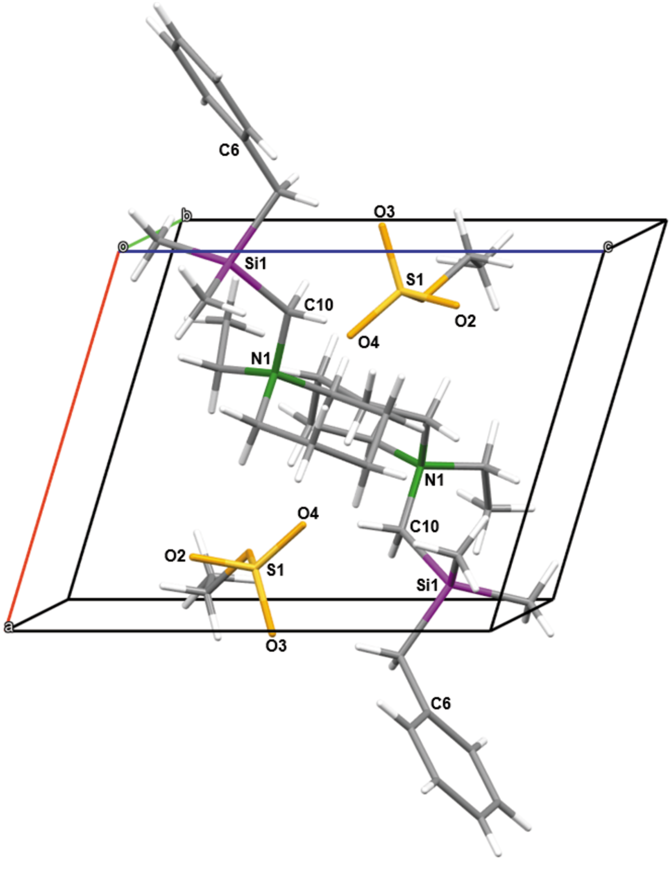

Figure 3
A view along the *b*-axis direction of the crystal packing of compound **1**.

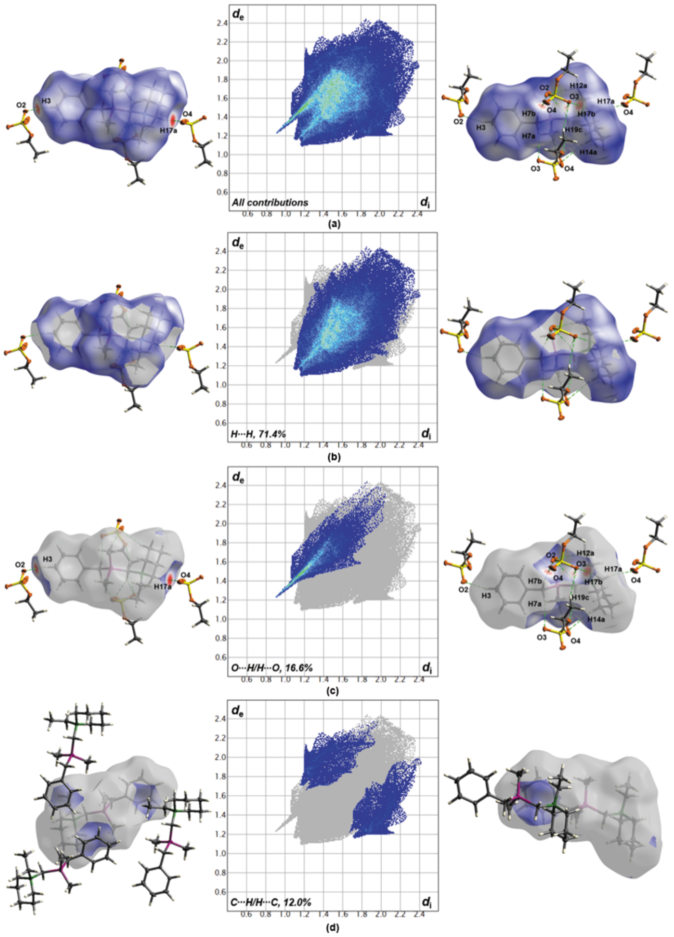

Figure 4
Hirshfeld surfaces and two-dimensional fingerprint plots of **1** showing close contacts for (*a*) all contributions in the crystal and (*b*) H···H, (*c*) O···H/H···O and (*d*) C···H/H···C interactions. Symmetry code: $-x, \frac{1}{2}+y, -z$.

4. Database survey

There are some other examples of crystallographically characterized α-aminosilane derivatives that are structurally based on compound **1** and its starting compound **2**. Examples of such α-piperidinosilanes found in the Cambridge Structural Database (WebCSD, November 2021; Groom *et al.*, 2016) are (*R*)-1-methyl-1-{[methyl(phenyl)(trimethylgermyl)silyl]methyl}-piperidinium iodide, $C_{17}H_{32}GeNSiI$ (CSD refcode BOFLOY; Strohmann *et al.*, 2008), (triphenylsilylpiperidinylcarbene)-tetracarbonyltungsten(0), $C_{28}H_{25}NO_4SiW$ (DIZWUE; Schubert *et al.*, 1986), [bis(trimethylsilyl)methyl]bis[diphenyl(*N*-piperidinomethylsilyl)methyl]gallium *n*-pentane solvate, $C_{45}H_{67}GaN_2Si_4{\cdot}0.5(C_5H_{12})$ (MASLUN; Uhl *et al.*, 2000), 8-chloro-8,8-dimethyl-1-aza-7-oxa-8-silabicyclo(4.3.0)non-6-ene, $C_8H_{16}ClNOSi$ (FUSYIB; Macharashvili *et al.*, 1987), 1-{[benzyl(methyl)phenylsilyl]methyl}piperidinium bromide, $C_{20}H_{28}NSiBr$ (NUPMUI; Barth *et al.*, 2015), *N*-(triphenylsilylmethyl)-5,6-aza-C60fulleroid, $C_{79}H_{17}NSi$ (YOXBOD; Hachiya *et al.*, 2009).

5. Synthesis and crystallization

The reaction scheme for the synthesis of **1** is illustrated in Fig. 5: 1-[(benzyldimethylsilyl)methyl]piperidine (**2**) (0.81 mmol) was dissolved in acetone (3 ml) and diethyl sulfate (0.81 mmol) was added dropwise to the solution. The reaction mixture was stirred and heated for 6 h at 329 K. Afterwards the reaction was quenched by the addition of a mixture of H_2O (2 ml) and NH_3 (2 ml). The aqueous phase was extracted three times with CH_2Cl_2 and the combined organic phases were dried over Na_2SO_4. After the removal of volatile compounds, the raw product was dissolved in *n*-pentane (1 ml) and stored at 243 K. The title salt (**1**) was isolated as colorless crystalline blocks.

^{1}H NMR (300.25 MHz, $CDCl_3$): δ = 0.30 [*s*, 6H, Si(CH_3)$_2$], 1.24–1.31 (*m*, 6H, OCH_2CH_3, NCH_2CH_3), 1.65–1.90 [*br. m*, 6H, N(CH_2CH_2)$_2$, $NCH_2CH_2CH_2$], 2.29 (*s*, 2H, SiCH_2C$_{ar}$), 3.12 (*s*, 2H, SiCH_2N), 3.37–3.56 [*br. m*, 6H, N(CH_2)$_3$], 4.12 (*q*, 2H, $^3J_{H-H}$ = 7.1Hz, OCH_2CH$_3$), 7.04 (*d*, 2H, $^3J_{H-H}$ = 7.0Hz, CH_{ar}), 7.10–7.15 (*m*, 1H, CH_{ar}), 7.24 (*d*, 2H, $^3J_{H-H}$ = 7.6Hz, CH_{ar}) ppm.

6. Refinement

Crystal data, data collection and structure refinement details are summarized in Table 3. All H atoms were refined freely using independent values for each U_{iso}(H).

Acknowledgements

J-LK and CS would like to thank the Fonds der Chemischen Industrie for a doctoral fellowship.

Funding information

Funding for this research was provided by: Verband der Chemischen Industrie.

References

Allen, F. H., Kennard, O., Watson, D. G., Brammer, L., Orpen, A. G. & Taylor, R. (1987). *J. Chem. Soc., Perkin Trans. 2*, pp. 1–19.
Barth, E. R., Golz, C., Koller, S. G. & Strohmann, C. (2015). *Acta Cryst.* E**71**, o759.
Bauer, J. O. & Strohmann, C. (2014). *Angew. Chem. Int. Ed.* **53**, 8167–8171.
Bent, H. A. (1960). *J. Chem. Educ.* **37**, 616–624.
Bent, H. A. (1961). *Chem. Rev.* **61**, 275–311.
Bruker (2021). *APEX4*, *SAINT* and *SADABS*. Bruker AXS Inc., Madison, Wisconsin, USA.

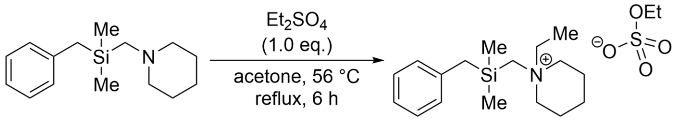

Figure 5
Reaction scheme of the alkylation of **2** with diethyl sulfate for the synthesis of **1**.

Table 3
Experimental details.

Crystal data	
Chemical formula	$C_{17}H_{30}NSi^+{\cdot}C_2H_5O_4S^-$
M_r	401.63
Crystal system, space group	Monoclinic, $P2_1$
Temperature (K)	100
a, b, c (Å)	8.4627 (8), 12.8187 (11), 10.3926 (9)
β (°)	107.033 (3)
V (Å^3)	1077.95 (17)
Z	2
Radiation type	Mo $K\alpha$
μ (mm^{-1})	0.23
Crystal size (mm)	0.82 × 0.44 × 0.38
Data collection	
Diffractometer	Bruker D8 VENTURE
Absorption correction	Multi-scan (*SADABS*; Bruker, 2021)
T_{min}, T_{max}	0.699, 0.747
No. of measured, independent and observed [$I > 2\sigma(I)$] reflections	68418, 8122, 7987
R_{int}	0.021
(sin $\theta/\lambda)_{max}$ (Å^{-1})	0.766
Refinement	
$R[F^2 > 2\sigma(F^2)]$, $wR(F^2)$, S	0.024, 0.065, 1.05
No. of reflections	8122
No. of parameters	375
No. of restraints	1
H-atom treatment	All H-atom parameters refined
$\Delta\rho_{max}$, $\Delta\rho_{min}$ (e Å^{-3})	0.53, −0.59
Absolute structure	Flack x determined using 3811 quotients $[(I^+)-(I^-)]/[(I^+)+(I^-)]$ (Parsons *et al.*, 2013)
Absolute structure parameter	−0.005 (6)

Computer programs: *APEX4* and *SAINT* (Bruker, 2021), *SHELXS* (Sheldrick, 2008), *SHELXL* (Sheldrick, 2015), *OLEX2* (Dolomanov *et al.*, 2009), *CrystalExplorer21* (Spackman *et al.*, 2021; Turner *et al.*, 2017), *publCIF* (Westrip, 2010), *Mercury* (Macrae *et al.*, 2020), *GaussView 6.016* (Frisch *et al.*, 2016), *Gaussian 09 Revision A.02* (Frisch *et al.*, 2016), *SCHAKAL99* (Keller, 1999) and *Molekel 4.3* (Flükiger *et al.*, 2000).

Denmark, S., Baird, J. D. & Regens, C. S. (2007). *J. Org. Chem.* **73**, 1440–1455.
Denmark, S. & Liu, J. H.-C. (2010). *Angew. Chem. Int. Ed.* **49**, 2978–2986.
Ditchfield, R., Hehre, W. F. & Pople, J. A. (1970). *J. Chem. Phys.* **54**, 724–728.
Dolomanov, O. V., Bourhis, L. J., Gildea, R. J., Howard, J. A. K. & Puschmann, H. (2009). *J. Appl. Cryst.* **42**, 339–341.
Etter, M. C., MacDonald, J. C. & Bernstein, J. (1990). *Acta Cryst.* B**46**, 256–262.
Flack, H. D. (1983). *Acta Cryst.* A**39**, 876–881.
Flükiger, P., Lüthi, H. P., Portmann, S. & Weber, J. (2000). *MOLEKEL 4.3.* Swiss Center for Scientific Computing, Manno , Switzerland.
Frisch, M. J., Trucks, G. W., Schlegel, H. B., Scuseria, G. E., Robb, M. A., Cheeseman, J. R., Scalmani, G., Barone, V., Petersson, G. A., Nakatsuji, H., Li, X., Caricato, M., Marenich, A., Bloino, J., Janesko, B. G., Gomperts, R., Mennucci, B., Hratchian, H. P., Ortiz, J. V., Izmaylov, A. F., Sonnenberg, J. L., Williams-Young, D., Ding, F., Lipparini, F., Egidi, F., Goings, J., Peng, B., Petrone, A., Henderson, T., Ranasinghe, D., Zakrzewski, V. G., Gao, J., Rega, N., Zheng, G., Liang, W., Hada, M., Ehara, M., Toyota, K., Fukuda, R., Hasegawa, J., Ishida, M., Nakajima, T., Honda, Y., Kitao, O., Nakai, H., Vreven, T., Throssell, K., Montgomery, J. A. Jr, Peralta, J. E., Ogliaro, F., Bearpark, M., Heyd, J. J., Brothers, E., Kudin, K. N., Staroverov, V. N., Keith, T., Kobayashi, R., Normand, J., Ragha-

vachari, K., Rendell, A., Burant, J. C., Iyengar, S. S., Tomasi, J., Cossi, M., Millam, J. M., Klene, M., Adamo, C., Cammi, R., Ochterski, J. W., Martin, R. L., Morokuma, K., Farkas, O., Foresman, J. B. & Fox, D. J. (2016). *Gaussian 09*, Revision A. 02. Gaussian, Inc., Wallingford, CT, USA.

Groom, C. R., Bruno, I. J., Lightfoot, M. P. & Ward, S. C. (2016). *Acta Cryst.* B**72**, 171–179.

Hachiya, H., Kakuta, T., Takami, M. & Kabe, Y. (2009). *J. Organomet. Chem.* **694**, 630–636.

Keller, E. (1999). *SCHAKAL99*. University of Freiburg, Germany.

Koller, S. G., Bauer, J. O. & Strohmann, C. (2017). *Angew. Chem. Int. Ed.* **56**, 7991–7994.

Krupp, A., Wegge, J., Otte, F., Kleinheider, J., Wall, H. & Strohmann, C. (2020). *Acta Cryst.* E**76**, 1437–1441.

Li, Y. & Hu, J. (2007). *Angew. Chem. Int. Ed.* **46**, 2489–2492.

Macharashvili, A. A., Baukov, Y. I., Kramarova, E. P., Oleneva, G. I., Pestunovich, V. A., Struchkov, Y. T. & Shklover, V. (1987). *Zh. Strukt. Khim.* **28**, 114–115.

Macrae, C. F., Sovago, I., Cottrell, S. J., Galek, P. T. A., McCabe, P., Pidcock, E., Platings, M., Shields, G. P., Stevens, J. S., Towler, M. & Wood, P. A. (2020). *J. Appl. Cryst.* **53**, 226–235.

Otte, F., Koller, S. G., Cuellar, E., Golz, C. & Strohmann, C. (2017). *Inorg. Chim. Acta*, **456**, 44–48.

Parsons, S., Flack, H. D. & Wagner, T. (2013). *Acta Cryst.* B**69**, 249–259.

Perlstein, J. (2001). *J. Am. Chem. Soc.* **123**, 191–192.

Schubert, U., Hepp, W. & Müller, J. (1986). *Organometallics*, **1986**, 5, 173–175.

Sheldrick, G. M. (2008). *Acta Cryst.* A**64**, 112–122.

Sheldrick, G. M. (2015). *Acta Cryst.* C**71**, 3–8.

Spackman, P. R., Turner, M. J., McKinnon, J. J., Wolff, S. K., Grimwood, D. J., Jayatilaka, D. & Spackman, M. A. (2021). *J. Appl. Cryst.* **54**, 1006–1011.

Strohmann, C., Bindl, M., Fraass, V. C. & Hörnig, J. (2004). *Angew. Chem. Int. Ed.* **43**, 1011–1014.

Strohmann, C. & Däschlein, C. (2008). *Organometallics*, **27**, 2499–2504.

Tomooka, K., Nakazaki, A. & Nakai, T. (2000). *J. Am. Chem. Soc.* **122**, 408–409.

Turner, M. J., McKinnon, J. J., Wolff, S. K., Grimwood, D. J., Spackmann, P. R., Jayatilaka, D. & Spackman, M. A. (2017). *CrystalExplorer17*. University of Western Australia.

Uhl, W., Cuypers, L., Schüler, K., Spies, T., Strohmann, C. & Lehmen, K. (2000). *Z. Anorg. Allg. Chem.* **626**, 1526–1534.

Westrip, S. P. (2010). *J. Appl. Cryst.* **43**, 920–925.

Zhao, Y. & Truhlar, D. G. (2008). *Theor. Chem. Acc.* **120**, 215–241.
